# Use, perceptions, and benefits of automotive technologies among aging drivers

**DOI:** 10.1186/s40621-016-0093-4

**Published:** 2016-12-19

**Authors:** David W. Eby, Lisa J. Molnar, Liang Zhang, Renée M. St. Louis, Nicole Zanier, Lidia P. Kostyniuk, Sergiu Stanciu

**Affiliations:** 1 University of Michigan Transportation Research Institute (UMTRI), 2901 Baxter Rd., Ann Arbor, MI 48109 USA; 2 Center for Advancing Transportation Leadership and Safety (ATLAS Center), Ann Arbor, MI USA; 3 Tsinghua University, Beijing, China

**Keywords:** Aging, Mobility, Automobile, Safety

## Abstract

Advanced in-vehicle technologies have been proposed as a potential way to keep older adults driving for as long as they can safely do so, by taking into account the common declines in functional abilities experienced by older adults. The purpose of this report was to synthesize the knowledge about older drivers and advanced in-vehicle technologies, focusing on three areas: use (how older drivers use these technologies), perception (what they think about the technologies), and outcomes (the safety and/or comfort benefits of the technologies). Twelve technologies were selected for review and grouped into three categories: crash avoidance systems (lane departure warning, curve speed warning, forward collision warning, blind spot warning, parking assistance); in-vehicle information systems (navigation assistance, intelligent speed adaptation); and other systems (adaptive cruise control, automatic crash notification, night vision enhancement, adaptive headlight, voice activated control). A comprehensive and systematic search was conducted for each technology to collect related publications. 271 articles were included into the final review. Research findings for each of the 12 technologies are synthesized in relation to how older adults use and think about the technologies as well as potential benefits. These results are presented separately for each technology. Can advanced in-vehicle technologies help extend the period over which an older adult can drive safely? This report answers this question with an optimistic “yes.” Some of the technologies reviewed in this report have been shown to help older drivers avoid crashes, improve the ease and comfort of driving, and travel to places and at times that they might normally avoid.

## Review

The fact that our society is aging and the potentially negative impacts this will have on safe transportation are well-known. In an effort to address older driver safety, it has been proposed that advanced in-vehicle technologies could be optimized to take into account the common declines in functional abilities experienced by older adults, thereby increasing their driving safety and comfort (see e.g., Band & Perel 2007; Eby & Molnar 2014; Marshall et al. 2014; Meyer 2009; Paris et al. 2014).

The purpose of this paper is to synthesize knowledge about older drivers and advanced in-vehicle technologies, with an overarching goal to address the question: Can advanced in-vehicle technology help extend safe driving? Specifically, this synthesis focuses on information about how older drivers use these technologies, what they think about the technologies, and the safety and/or comfort benefits of the technologies. This synthesis is limited to manufacturer-installed advanced technologies. We excluded electronic stability control (ESC) because the safety benefits are established (see e.g., Chouinard & Lècuyer 2011; Dang 2007; Ferguson 2007; Sivinski 2011), the technology is unobtrusive to drivers, and it has been required on all light vehicles since 2012 (National Highway Traffic Safety 2015). Vehicle design issues and advanced crashworthiness technologies are also not covered. Finally, the review excludes autonomous vehicle (also called “driverless car” and “self-driving car”) technologies.

## Methods

The search for literature on advanced in-vehicle technologies and older drivers entailed a number of steps. First, we developed a list of the new advanced in-vehicle technologies that might benefit older drivers based on previous research (Eby & Molnar 2014; Eby et al. 2009; Mulholland 2009; The Hartford 2012) and the expertise of the research team. Based on this, 12 technologies were selected and grouped into three categories as shown in Table 1. Note that four other technologies (intersection assistance, merging assistance, congestion warning, and drowsiness/fatigue warnings) were also included in the review, but are not reported here because these systems are still early in development or are prototypes. A synthesis of these technologies is available in another report (Eby et al. 2015).

**Table 1:** Categorization of advanced vehicle technologies and records of literature search

Category	Advanced In-Vehicle Technology	Number of articles identified	Number of articles reviewed
Crash avoidance systems	Lane departure warning/mitigation (LDW)	821	29
	Curve speed warning (CSW)	144	4
	Forward collision warning/mitigation (FCW)	134	29
	Blind spot warning (BSW)	274	22
	Parking assistance (PA)	140	25
In-vehicle information systems	Navigation assistance (NA)	139	27
	Intelligent speed adaptation (ISA)	316	31
Other systems	Adaptive cruise control (ACC)	153	38
	Automatic crash notification (ACN)	173	12
	Night vision enhancement (NVE)	78	13
	Adaptive headlight (AH)	250	23
	Voice activated control (VAC)	173	18
Total		2795	271

Publications on each of these technologies were searched comprehensively in SCOPUS, TRID, and DEEPBLUE (a digital repository of University of Michigan reports). From these databases, we gathered relevant journal articles, conference papers, technical reports, and books with the restriction that they be published in English. To aid in finding articles related to these technologies and older drivers, search terms were developed for three parameters: the various names for each technology (e.g., lane departure warning system, lane keeping system, lane keeping assistance; lane keeping support system); terms used to describe the older adult population (e.g., older, elderly, aged, aging, senior, and mature); and terms that restricted results to the driving domain (e.g., driving, driver, vehicle, and automobile). For some technologies for which there was little literature, we did not restrict our search to only older adults. In addition, the search was not restricted by year, but most studies found in the review were published in the 1990s onward. Manual searches of the reference lists of selected key articles were also conducted to collect additional relevant articles. As shown in Table 1, the initial search yielded 2795 articles.

Articles produced from this search were then reviewed for appropriateness. To be included for further review, the study had to meet several criteria: 1) be related to how older drivers use, think about, or are affected by the specific in-vehicle technologies; 2) focus on the safety and mobility benefits of these technologies, rather than the environment, congestion, or other benefits; and 3) either address older drivers specifically or include older drivers as part of the larger population being addressed. Due to a general lack of studies utilizing an older driver group, this criterion was relaxed for some technologies for which associated studies included more than just young drivers. This review process yielded a total 308 articles. The number of articles per technology is shown in Table 1. Because 37 articles addressed more than one technology, the actual number of unique articles was 271. The articles represented a wide range of research methods including questionnaires, focus groups, structured interviews, crash record analysis, naturalistic driving, and simulated driving. Relevant articles for each technology were reviewed and the knowledge was synthesized by the authors.

## Results

The results are presented by the three categories of advanced in-vehicle technologies outlined earlier: crash avoidance systems; in-vehicle information systems; and other systems. It is important to note that this categorization is somewhat artificial and that some of the technologies fit into more than category. For example, parking assistance technologies span the range of assistance from simply providing a rear-view camera to automatically parallel parking a vehicle. Technologies in the first group would be categorized as in-vehicle information systems while technologies in the latter group would be categorized as crash avoidance systems given that they can help prevent a crash. In these cases we simply chose a category for the technology and synthesized all of the information for that technology in one place.

### Crash avoidance systems

One particularly promising category of technologies involves systems that directly target the prevention of crashes. These systems, collectively called crash avoidance systems, use on-vehicle radars, cameras, other sensors, and computer intelligence to determine the situations that could lead to a crash. When a potentially hazardous situation arises, the system either provides a warning to the driver that an action may be required, or takes over temporary control of an operational aspect of the vehicle (such as braking) and engages that system without driver input to avoid a crash. This section reviews the following technologies: lane departure warning systems; curve speed warning systems; forward collision warning systems; blind spot warning; parking assistance systems; intersection assistance systems; and merging assistance systems.

#### Lane departure warning/mitigation

A number of technologies have been developed in recent years that are designed to keep a driver from inadvertently driving outside of a travel lane, thereby assisting the driver in proper lane keeping behavior and ultimately preventing run-off-the-road crashes. Lane departure warning/mitigation (LDW) systems (also called Lane Keeping Assistance and Lateral Drift Warning Systems) utilize video camera and image analysis software to determine the location of lane markings relative to the vehicle (LeBlanc et al. 2006). When the vehicle drifts too close to the markings without a turn-signal activated, the driver is given some form of an alert that is most often directionally-linked such that a drift to the left is accompanied by a visual (e.g., flashing icon), auditory (e.g., beep), or haptic (e.g., slight steering wheel force in the direction away from the drift) warning on the driver’s left side. In some cases, the system can also take partial control of the vehicle to help maintain proper lane position. For example, some commercially-available systems, in addition to the warning, can also apply slight brake pressure to the wheel opposite the lane departure to help move the vehicle back into the center of the lane (Braitman et al. 2010).

A number of studies have estimated the safety benefits of LDW systems under the scenario that the light-vehicle fleet in the US was equipped with the systems and all drivers used them. Under a variety of assumptions, the safety benefits of LDWs have been estimated across the entire US population of drivers as leading to an overall reduction in all crashes of about 3 %, all lane-departure-related crashes of about 30 %, lane-departure crashes with serious injury of about 25 %, and lane-departure fatal crashes of about 10–20 % (Blower 2014; Jermakian 2011; Kusano & Gabler 2014; Kusano et al. 2014). Studies looking specifically at the estimated crash reduction benefits among the older population have not been published.

Little research has addressed older drivers’ use of LDW systems. A number of studies that utilized simulators have investigated the effectiveness of various types of alerts to help drivers respond appropriately to the warning (Cummings et al. 2007; Deroo et al. 2012; Edwards et al. 2013; Kozak et al. 2006; Navarro et al. 2007; Suzuki & Jansson 2003). None of these studies focused specifically on older adults. Collectively, the results showed that haptic warnings (particularly small pulses to the steering wheel in the direction of the center of the travel lane) accompanied by an auditory warning, resulted in the fastest and most accurate driver response. Warnings that were directionally-linked were more effective than those that were not. These results would likely hold for older adults, but this should be confirmed in further research.

Studies making use of instrumented vehicles on actual roadways also shed light on the safety benefits of LDW systems. One such study in Germany investigated the lane keeping performance among 30 drivers (some as old as 65 years) who were asked to dial a phone while driving a vehicle equipped with a LDW system (Blaschke et al. 2009). The study found significantly better lane keeping while dialing the phone when the LDW system was providing alerts (steering wheel vibration in this case). Departures from the travel lane were found only in the conditions where the LDW was turned off. The LDW system was also judged to be helpful by the participants. No analyses by age were presented.

In an investigation of 78 people (26 of which were aged 60–70) in Michigan using a LDW system in over 83,000 miles of driving, LeBlanc and colleagues (LeBlanc et al. 2006) found that the system induced drivers to stay closer to the center of the lane, use their turn signals more often when changing lanes, and reduced the frequency of lane excursions. When compared to younger age groups, the older group in the study judged the LDW system to be more useful.

The assessment of LDW systems among older drivers has been mixed in other global studies that have utilized focus group and interview techniques. A focus group study in Australia (Regan et al. 2002) found that older drivers (age 65 and older): thought that LDW systems would be useful, especially for long trips; expressed uncertainty about the effectiveness of LDW systems, particularly in various weather and road conditions; were concerned about whether the system could give them a warning that was early enough for them to take a corrective action; and expressed some concern that the system would lead to distraction. A German study that interviewed 32 drivers age 60 to 80 years who owned vehicles equipped with driver assistance systems (not necessarily a LDW system) expressed moderate concerns about the effectiveness and usefulness of LDW systems, but most had little actual experience with these systems (Trübswetter & Bengler 2013). In a focus group study in Sweden of drivers age 39 to 74 who drove vehicles equipped with some form of advanced driver assistance system, participants indicated that they used their turn signals more often with the LDW system, but expressed concern about the system not working in all driving conditions (Strand et al. 2011).

A study in the US interviewed 183 owners of vehicles equipped with LDW systems (Eichelberger & McCartt 2014a). Nearly half of the participants were age 61 and older. This study found that 71 % of participants wanted an LDW system on their next vehicle and most reported that they drove more safely when using the LDW system. Finally, another US study interviewed 301 drivers of vehicles with LDW warnings only (10 % were age 61 and older) and 184 drivers of vehicles with LDW that also actively helped to steer the vehicle back to the center of the lane, where 19 % were age 61 and older (Braitman et al. 2010). Of those drivers in the LDW-warning-only group: 69 % reported always used the system; 47 % reported receiving erroneous warnings, usually in situations where lane markings were poor or covered; 71 % reported that the system helped them with proper lane keeping; 75 % said the system made them a safer driver; 54 % reported using their turn signals more often; 34 % said the system relieved stress; and 41 % thought the system was annoying. Of the respondents who had a LDW prevention system (i.e., one that helped to steer back into the lane center) in their vehicle: 15 % always had the system turned on; 22 % were unaware that their vehicle had the system; and 22 % never used the system (note that this system defaulted to off and had to be turned on for each trip). Of those who had used the system: 10 % reported getting false or unnecessary warnings; 15 % thought the intervention component of the system was annoying; 68 % reported drifting less often in their lane; 64 % reported using their turn signals more often; and 83 % expressed that they would want the system again.

#### Curve speed warning

A system that is closely related to lane departure warning systems, are curve speed warning (CSW) systems which use global positioning system (GPS) information and digital maps to determine the risk associated with a vehicle approaching a curve at a certain speed and warns that driver if the approach speed is too fast for the curve (University of Michigan Transportation Research Institute 2015). Only a handful of studies have addressed CSW systems with older adults.

One of these studies compared 24 young drivers (age 18–25) to 24 older drivers (age 60 and older) on responses to combinations of three CSW alert types: visual (a flashing numeral that indicated the proper speed); auditory (a voice instructing the driver on the proper speed); and haptic (3-s force on the accelerator pedal against the driver’s foot) (McElheny et al. 2006). Drivers were tested at night on a closed driving course. Overall, drivers exhibited quicker reaction times and more appropriate speeds at curves when they received a CSW than in a baseline condition with no warning. The older drivers were significantly closer than the young drivers to the appropriate speed in response to the CSW. The older drivers were also significantly more likely to want a CSW system in their vehicle that included a visual-auditory-haptic set of warning types.

Another study tested a CSW system over a 1-month period in which 78 drivers (26 of whom were aged 60 and older) drove a test-vehicle equipped with both a CSW and LDW system in a natural setting (LeBlanc et al. 2006). The CSW system utilized a combination of visual (icon), auditory (message), and haptic (seat vibration) warnings. Overall, the CSW system did not significantly change objective curve-taking behaviors (analyses on this issue by age group were not presented). Participant ratings of the CSW system were generally positive, with older drivers giving slightly more positive ratings.

A final study investigated an integrated set of crash-avoidance technologies that included a CSW component (Sayer et al. 2010). One-hundred eight volunteers (36 of whom were age 61–69) drove a test vehicle for a 40-day period using it as their personal vehicle. During the final 30 days of the study, the crash avoidance technologies were operational. As with the previous study, there was no significant change in objective curve-taking behaviors either when approaching or when negotiating curves. Of the other components in the system, the CSW component was rated as one of the least useful. No analyses by age were reported for this component of the integrated system.

The limited research shows that older drivers like CSW systems, and in experimental tests, use of the systems resulted in older drivers taking turns at speeds that were closer to the recommended speed. However, studies that have assessed the impact of CSW systems on older adults’ driving behaviors under normal driving conditions have found no change in driving behaviors, despite the finding that older drivers liked CSW systems.

#### Forward collision warning/mitigation

Forward collision warning/mitigation (FCW) systems use forward radars and other sensors to determine the changing distances to vehicles and objects in front of the driver’s vehicle. When the system determines that the vehicle is in danger of colliding with the forward obstacle, the system will warn the driver using some signal (usually a combination of a light and sound) and, in some systems, take over partial control (e.g., braking) of the vehicle. FCW has great potential for preventing crashes and the associated death and injuries. Nationally, studies estimated that with full-market penetration, FCW systems could prevent up to 20 % of all crashes (Blower 2014; Jermakian 2011; Kusano & Gabler 2014). This translates into an annual reduction of 1.2 million crashes, 66,000 non-fatal injuries, and 879 fatalities (Jermakian 2011). Unfortunately, there are no estimates of crash reduction by age of driver.

Several studies have investigated the use, perceptions, and benefits of FCW systems utilizing driving simulators in scenarios that involve high risk for a frontal crash, such as a lead vehicle suddenly braking. These studies have found that: FCW systems reduced crash likelihood; driver acceptance was high when the system did not give too many false alarms (giving alerts when they were not appropriate); older drivers were more forgiving of false alarms when they understood the cause of the false alarm; older drivers reacted just as quickly to collision events as younger drivers, even though the older participants had slower reaction times in a laboratory setting; and older participants drove more slowly than younger drivers and maintained longer headways from the next vehicle when using the system (Cotté et al. 2001; Kramer et al. 2007; Maltz & Shinar 2004).

These systems were also tested on roadways (either on closed test-track or on actual roads) with instrumented vehicles in several studies. In a study on a closed test-track with an instrumented vehicle equipped with a FCW system, researchers found that older participants maintained longer headways when compared to young drivers, similar to what was found with the simulator studies. Further, the headways for older participants did not change as false alarms increased, indicating that older drivers were more tolerant of false alarms (Dingus et al. 1997). A study in Italy tested a FCW system in controlled drives in real city traffic utilizing an instrumented vehicle equipped with a FCW system (Adell et al. 2011). This study included 20 drivers, 10 of whom were age 45–69. The study found that drivers reacted more quickly to threats, drove with longer headways, and were better able to detect pedestrians in the roadway while using the system. On the negative side, when using the system drivers had significantly more center lane crossings and harder braking at traffic lights. No other safety-related driving behaviors were impacted.

Two large-scale field operational tests have evaluated FCW systems with drivers under natural driving conditions (Sayer et al. 2010; Ervin et al. 2005; LeBlanc et al. 2013). One third of the roughly 100 participants in each study were age 60–70. In both studies, participants drove instrumented vehicles that were equipped with a FCW system (and other crash avoidance systems) for 1 week with the system turned off and then 3 additional weeks with the system operational. Participants were instructed to drive as they normally would. The instrumented vehicles recorded a wide range of measures automatically as participants drove. Collectively, these studies found: when compared to not using the system, the FCW system improved safety for all drivers and did not impact other safety behaviors such as engaging in more frequent secondary tasks while driving; older participants were more likely to view the system favorably, although most judged the system usefulness as neutral; older participants drove with more distance from the lead vehicle than participants in other age groups; many drivers reported receiving alerts that were not necessary; and many drivers thought that the system would improve safety, but generally this perception was directed at other age groups rather than their own.

Several studies have conducted focus groups and interviews with drivers (the percent of participants age 60 and older in each study ranged from 26 to 56 %) who had a FCW system on their personal vehicles (Braitman et al. 2010; Strand et al. 2011; Eichelberger & McCartt 2014a; Cicchino & McCartt 2014; Eichelberger & McCartt 2014b). The results of these subjective studies are fairly consistent and show: a large majority of drivers (84 to 97 %) always kept the system on; 40 to 55 % of drivers had received alerts from the system and about one-half thought the system helped to prevent a crash; slightly more than one-half of respondents reported that the system never failed to warn them of a crash, but a larger percentage also believed they received false alarms; and participants generally reported that the system made them more aware of following distances and some reported driving with a greater following distance when using the system.

There is overwhelming evidence attesting to the safety benefits of FCW systems for all drivers and for older drivers specifically. For older adults, the evidence shows that FCW systems can help prevent crashes without causing a negative impact on other behaviors such as increased speeding or more frequent engagement in non-driving tasks. Older drivers who have used these systems under normal driving conditions are favorable toward them and a large majority reported that such systems had prevented crashes. There is some concern about false alarms among older adults that should be addressed in future designs.

#### Blind spot warning

To safely change lanes, it is important for a driver to ensure that the lane he or she intends to enter is not blocked by another vehicle, bicycle rider, or other obstacle, through a direct visual search of the area surrounding the vehicle and a scan of the mirrors. If the driver does not turn his or her head to check for traffic, there are areas around the vehicle where objects cannot be seen even in side-view mirrors (the blind spot), putting the driver at higher risk of crash. A simulator study comparing blind spot checking among younger (age 21–31) and older (age 65–75) drivers found that the older drivers checked blind spots significantly less frequently (41 % versus 86 %) and older drivers turned their heads less far when they did check the blind spot (Lavallière et al. 2011a). A study using an instrumented vehicle under actual highway driving conditions found that older drivers were less than half as likely (24 % versus 53 %) to check the blind spot during a lane change (Lavallière et al. 2011b).

Blind sport warning (BSW) systems use radars or cameras to monitor the location of traffic or obstacles in a vehicle’s blind spot zones and provide warnings to the driver about these obstacles during a lane change maneuver (Jermakian 2011; Kessler et al. 2012). As with most collision avoidance systems, these warnings can be visual, auditory, or haptic (see e.g., (Chun et al. 2013; Guo et al. 2010)). These systems are also called side-view assist, blind spot monitor, and lane change/merge assist. BSW systems are designed to help prevent lane-change-related crashes. According to one estimate, if all US vehicles were equipped with BSW systems, about 20,000 moderate-to-severe injuries and 393 fatal crashes each year could be prevented (Jermakian 2011).

A European study that examined safety-related behaviors of middle-age drivers while using a BSW system found that turn-signal use decreased significantly (about 10 %) when compared to the same drivers not using the system (Kessler et al. 2012). A simulator study also found a decreased use of turn signals among older adults (Guo et al. 2010). This result seems counterintuitive, but subjective data from these studies showed that drivers trusted the system to let them know if a vehicle was in the intended lane, so the turn signal did not seem as important. When a BSW system is combined with a LDW system, however, turn signal use has been shown to increase (Sayer et al. 2010). This same study, which included an older adult age group, found that when using the BSW system, participants adjusted their lane position slightly away from vehicles in the blind spot, indicating that the BSW increased driver awareness of adjacent traffic. Test-track results with middle-age participants indicated that a BSW system helped drivers react more quickly to a lateral crash threat and drivers reported that they liked receiving the BSW alerts (Fitch et al. 2014). A study that utilized test vehicles with a BSW system being driven in actual traffic with drivers age 40–70 found that mirror-checking prior to a lane-change significantly increased (Kiefer & Hankey 2008).

Several studies have reported subjective impressions of a variety of BSW systems among older drivers. Collectively, these studies have found: there are concerns about the system accuracy and false alarms, particularly in bad weather; mixed results about whether older adults would want the system in a future car; and some older adults reported that the system could be distracting (Braitman et al. 2010; Trübswetter & Bengler 2013; Strand et al. 2011; Cicchino & McCartt 2014). Findings from large-scale interview studies of drivers (about 30 % of whom were age 61 or older) with BSW systems in their personal vehicle indicated that: the system had helped to prevent a lane-change crash; the system was used frequently; high levels of system reliability were reported but also there were frequent reports of false warnings, generally during bad weather; little change in turn signal or mirror use; less frequent turning of the head to check a blind spot in about one-third of participants; and the system made users feel safer and less stressed (Braitman et al. 2010; Cicchino & McCartt 2014).

Given the difficulties that many older drivers have with turning their heads to check the areas around their vehicles, BSW systems could have significant value for older drivers. Studies with older drivers have shown not only that the systems have prevented crashes, but also that their use can promote more frequent mirror checking and increase situational awareness. Some work has found a decreased use of turn signals among older adults using a BSW system, however when the system is combined with a LDW system, turn signal use has been shown to increase for this age group. Some results with older drivers also suggest that this group may place too much trust or be overconfident in the system and that BSW systems could increase distraction. This suggests that training on BSW systems would be useful for older drivers. False alarms during bad weather are also reported by older drivers, indicating the need for better future designs or the ability to turn off the system under conditions in which it performs poorly.

#### Parking assistance

An unavoidable component of driving is the need to park. Studies have long shown that many older drivers have difficulty parking and often rate parking, particularly parallel parking, as stressful (Baldock et al. 2006; Douissembekov et al. 2014; Herriotts 2005; Lyman et al. 2001; Parker et al. 2001; Stalvey & Owsley 2000). For example, one study found that 37 % of older drivers avoided parallel parking at least some of the time, with 11 % indicating that they always avoided parallel parking (Stalvey & Owsley 2000). Backing out of a parking space or a driveway can also be dangerous. According to NHTSA (National Highway Traffic Safety Administration 2006) estimates, there were 183 fatalities and about 6700 injuries caused by back-up crashes, with older drivers having the highest involvement rate for this type of crash per licensed driver.

Technologies called parking assist (PA) systems have been developed to assist drivers in a number of parking-related tasks, including parallel parking, backing into a perpendicular parking spot, and backing out of a parking space. We include these technologies under the section on collision avoidance systems because they can help prevent collisions with obstacles and cross-traffic when leaving a parking space. However, these systems are also designed to make parking easier and less stressful. Here we review three general types of systems: backup cameras and obstacle warning alerts; cross-traffic alerts; and semi-autonomous parallel parking.

Systems designed to help drivers back up typically include rear cameras that could show the driver the scene behind the vehicle on an in-vehicle video display. These systems can also include enhancements overlaid on the video output that, for example, show graphically the location where a vehicle will end up given the current direction of the front wheels. Some systems, either independently or in conjunction with a camera, provide alerts about obstacles behind the vehicle. Several studies have addressed the use, effectiveness, and perceptions of these systems. One study investigating glance behavior found that people of all ages rarely (8–20 %) looked at backup camera displays before backing up, but nearly one-half would look at the display after they were presented with an obstacle detection alert (Hurwitz et al. 2010). A naturalistic driving study of 37 drivers (age 25–60) of vehicles equipped with a rear camera (with and without obstacle detection) revealed that drivers look at rearview video displays during backing maneuvers at least some of the time, with approximately 10–14 % of glances going to the display while backing. In addition, no evidence was found that driver’s backing behavior (i.e. speed and acceleration) was influenced by this display (Mazzae et al. 2008). An experimental study of a backup assistance system that utilized rear video, obstacle detection, and auditory/visual information on distance to rear obstacles tested a number of use-measures in a set of parking/backup scenarios with 32 drivers who were age 45 and older (McLaughlin et al. 2003). The study found that: when compared to no backup system: participants parallel parked closer to the curb (8 cm on average); for backing into a perpendicular parking space, participants parked significantly closer to the back of the space; and participants were significantly better at aligning a trailer hitch to a trailer. Some studies have investigated the ability of systems with obstacle detection (with and without a rear camera) to prevent a collision with an obstacle (usually a traffic cone) placed behind the vehicle by an experimenter without the knowledge of the participant (Hurwitz et al. 2010; Mazzae et al. 2008; McLaughlin et al. 2003; Llaneras et al. 2011). These studies have found high rates of hitting the obstacle (over 80 %). However, when glance behavior was analyzed, only a small minority of drivers hit the obstacle if they had looked at the rearview display. Finally, experiences and impressions with backup assistance systems were collected in several studies from drivers who had these systems on their own vehicles. A recent study in the US collected subjective data from older drivers who owned vehicles equipped with backup obstacle detection systems, some which also provided distance-to-obstacle information (Cicchino et al. 2015). This study found that: nearly all drivers never turned the system off and received warnings at least once a week; 56 % reported that they had heard an alert and noticed an obstacle behind their vehicle for which they were previously unaware; 30 % reported that the system often provides alerts when there was nothing behind the vehicle; 95 % reported that they would want the system in their next vehicle; 55 % reported that the system relieved stress; and 1 % reported that the system was distracting. Other studies that have investigated older drivers’ impressions of backup assistance systems have found that: there is some confusion about how the systems operate; people thought the system would help them avoid crashes; and systems that included both a rear camera and an obstacle warning were more highly regarded (Hurwitz et al. 2010; Mazzae et al. 2008; McLaughlin et al. 2003; AAAFTS 2008).

A similar type of parking assist system utilizes camera and sensor technology to help identify the presence of cross-traffic and alerts the driver when he or she is backing out of a parking space and cross-traffic is present. In some cases, the system will brake automatically when cross-traffic is detected (see e.g., Seto et al. 2012). A US study in Massachusetts investigated the effects of a cross-traffic alert system in response to real vehicle encroachment with 42 drivers (one-third of whom were age 61–68) (Reimer et al. 2010). The study found that when compared to not using the system, participants experienced slightly but not significantly lower stress levels as measured by changes in heart rate. All participants using the system stopped when cross-traffic was approaching compared to 71 % who stopped appropriately when not using the system. The study also reported that there were false alarms on about 5 % of trials. Subjective ratings from this study showed that 79 % of participants reported that the system made them safer while backing up and 67 % reported that the system reduced the stress associated with backing up. A national study of 210 owners (one-third of whom were age 61 and older) of vehicles equipped with a cross-traffic warning system answered questions about the system (Cicchino & McCartt 2014). The study found that: the systems were turned on in nearly all vehicles (95 %) and nearly all drivers had experienced alerts from the system; three-fourths of drivers reported that they were always alerted when cross-traffic was present; 34 % reported getting unnecessary alerts, primarily in bad weather or when there were stationary objects off to the side; 75 % thought the system was useful for backing up; and most reported no changes in their backing up behavior in response to the system.

A third type of parking assist system (sometimes called semi-autonomous parking assist) takes over the steering component of maneuvering into a parallel parking space. Studies testing these types of systems in real-world settings with middle-age and older drivers have found that use of the system: reduced mental workload when parking (Tachibana 2011; Totzke et al. 2010); reduced stress as measured by a reduction in heart rate (Reimer et al. 2010); improved parking behavior as measured by a number of factors (Reimer et al. 2010; Totzke et al. 2011); rated more positively and might transfer learning so that parking will be improved even when the system is not being used (Reimer et al. 2010; Totzke et al. 2011; Kawabata et al. 2008).

Given that older drivers report parking and backing up to be difficult and stressful, technologies that help older adults do these tasks more safely and effectively would be welcome for this group. The research shows that backup cameras alone have no significant impact on backing up safely, primarily because older drivers tended not to look at them. Adding enhancements to the backup video display such as obstacle detection warnings or distance-to-obstacle information, however, does seem to help older adults notice obstacles behind the vehicle of which they were previously unaware, park more squarely in parking spaces, and reduce stress. PA systems that provide cross-traffic alerts seem like they would be valuable for older drivers, yet little research had addressed the use of these systems with them. Those studies that have utilized older drivers, have found little change in backing behavior and high levels of false alarms. More research with older adults is needed before cross-traffic alert systems can be recommended for older drivers. Older driver research on PA systems that take over the steering component of parallel parking have clearly shown that these systems provide numerous benefits for older adults, including a reduction in stress and mental workload and improved parking.

### In-vehicle information systems

In-vehicle information system (IVIS) technologies are designed to provide a driver with information that he or she can use to make better driving decisions (Simões & Pereira 2009). Generally this information is not intended for the second-by-second operation of the vehicle but rather for improving strategic driving decisions over a longer time-frame, such as deciding where to make a turn or preparing for upcoming traffic congestion. Here we review three IVISs—navigation assistance, congestion warnings, and intelligent speed adaptation.

#### Navigation assistance

Navigation assistance (NA) systems combine global positioning system (GPS) vehicle location information with digital map data to provide drivers with turn-by-turn, instructions (visual and auditory) to locations as they drive. Some systems can utilize cellular or other communication means to obtain real-time traffic volume information and adjust guidance information to avoid traffic congestion (see e.g., (Kostyniuk et al. 1997a)). Nomadic NA systems are widely available as an aftermarket product and most smartphones have applications that provide NA system functionality. This review, however, is limited to synthesizing the research on manufacturer-installed NA systems.

Given the difficulty older drivers have in wayfinding (see e.g., (Bryden et al. 2013)), particularly in unfamiliar areas, NA systems have been cited as being particularly helpful for older drivers (Band & Perel 2007; AAAFTS 2008; Baldwin 2002; Eby & Kostyniuk 1998; Eby & Kostyniuk 1999; Eby & Molnar 1999; Kostyniuk et al. 1997b). Several studies have assessed older drivers’ use and perceptions of NA systems under actual and simulated driving conditions. Collectively these studies showed that older drivers: used NA systems frequently; reported only minimal distraction, but more than reported by younger drivers; traveled to places they would not have gone to without the system; more frequently travelled during times and on roadways that they would normally avoid; reported increased feelings of safety, confidence, attentiveness, and relaxation when using NA systems; tended to still bring paper maps along in case the NA system failed; took longer and had more difficulty learning to use NA systems; were more likely to have learned how to use the system from a friend or family member; had more difficulty than younger drivers reading the displays; more frequently used the system with a “co-navigator” passenger than reported by younger drivers; some reported feeling that the NA system was more like a human co-navigator than a technological device; reported higher preferences for verbal turn-by-turn instructions; and would not buy a system targeted to “old” people (Dingus et al. 1997; Kessler et al. 2012; Eby & Kostyniuk 1998; Eby & Kostyniuk 1999; Eby & Molnar 1999; Chan & Rose 2002; Emmerson et al. 2013; Novotný & Bouchner 2011; Oxley et al. 1995; Vrkljan & Polgar 2007; Zhang et al. 2012)

Some older drivers report difficulty in wayfinding and many older adults report being uncomfortable driving in unfamiliar areas. An abundance of evidence suggests that NA systems provide many benefits to older drivers provided that the interfaces are easy-to-use and intuitive. NA systems are frequently used and highly regarded by older adults.

#### Intelligent speed adaptation

It is well-established that speeding is a causative factor in motor vehicle crashes (e.g., Liu et al. 2005; McGwin & Brown 1999; Siskind et al. 2011), with about 30 % of fatal crashes in the US attributed to speeding (National Highway Traffic Safety Administration 2012). Studies, however, have shown that speeding is an infrequent behavior among older adults and those with age-related medical conditions (Charlton et al. 2006; Eby et al. 2012). Older adults are also underrepresented in speed-related traffic crashes (McGwin & Brown 1999; Langford & Koppel 2006; Planek & Fowler 1971; Stamatiadis 1996).

In an effort to curtail speeding and the resulting crashes, technologies, called intelligent speed adaptation (ISA) systems, have been developed to encourage people to drive at the set speed limits. ISA systems use vehicle location information, driving speed, and an underlying database to determine the relationship between the vehicle speed and the speed limit for the road on which a driver is travelling (Marchau et al. 2010). If the driver is speeding, the systems are designed to give in-vehicle feedback (visual, auditory, and/or haptic) to the driver. Some systems can also take over partial control of the vehicle and decrease vehicle velocity to the posted speed limit. Some systems have also been linked with auto-enforcement units that can deliver fines and/or rewards. Several studies have concluded that if ISA systems were in wide-spread use, there would be a significant decrease in crashes, injuries, and the associated costs (see e.g., Carsten & Tate 2005; Doecke & Woolley 2011; Lai et al. 2012).

Simulator studies on the effects of ISA on the driving behaviors and perceptions of participants have generally not included older adults. These studies have found that: ISA systems that simply inform drivers that they are speeding do not change speeding or passing behavior; ISA systems that took over partial control of the vehicle reduced speeding, decreased following distance in some studies, and decreased passing behavior frequency; both types of systems were judged as useful and would improve road safety; and neither system was judged as satisfactory or desirable (Comte 2000; Jamson 2006; Jamson et al. 2012; Spyropoulou et al. 2014). A simulator study in Japan compared 15 young drivers to 16 older drivers (no ages given) on use of ISA systems and reactions to various types of ISA advisories (Ando et al. 2014). This study found that the advisories had a larger effect on young drivers due to the fact that younger drivers drove faster without the ISA system.

The effects of ISA system on driver behavior and attitudes have been investigated in real traffic in a number of studies, some of which included older adults (Lai & Carsten 2012; Lai et al. 2010; Reagan & Bliss 2013; Regan et al. 2006; Vlassenroot et al. 2007; Wall et al. 2013). When compared to not using a system, use of an ISA system that actively slowed the vehicle to prevent speeding shows that: the system significantly reduced excessive speeding; speed profiles were closer to posted speed limits; the system was overridden (turned off) by the driver most often on high speed roads (16 % of the time for 70 mph roads) followed by the lowest speed roads (13 % of the time for 20 mph roads); speeding was frequent when the system was overridden; and the longer the system was used the more frequently the system was overridden (Lai & Carsten 2012; Lai et al. 2010).

An ISA system in the US that provided auditory and visual advisories about speeding (differentiating between “moderate” and “egregious” speeding) was tested with 50 participants (age 25–35) in real traffic over a 1-month period (Reagan & Bliss 2013; Reagan et al. 2013). The study included three groups. One was a control group of 10 drivers who drove without the ISA system operating. One group drove with the system activated (feedback only). The final group of participants drove with the ISA activated but also was given a monetary incentive to drive within the speed limit. This latter group of participants were told that they could earn up to $25 at the end of the study, with the amount decreasing slightly with each instance of speeding (-$0.03 for moderate and -$0.06 for egregious speeding). The study found that: there was a moderate reduction in speeding with the feedback only and a significant reduction in speeding with the incentive; mental workload increased for both systems; the system was rated positively for reliability, predictability, accuracy, agreeableness, trustworthiness, and acceptability; participants found the auditory component of the system annoying; and participants would not pay to have the system in their vehicle.

Questionnaire studies in several countries have explored the general public’s opinion of ISA systems, including older adults (Chorlton et al. 2012; Eriksson & Bjørnskau 2012; Garvill et al. 2003; Vlassenroot et al. 2010; Warner et al. 2010). Collectively, these studies have found: agreement that speeding is an important traffic safety issue; limited awareness of or experience with ISA systems; moderate support for a voluntary ISA system that provided feedback only; low support for ISA systems that actively control speed; moderate support for ISA systems being used only on certain roadways or in the vehicles of frequent speeders; and that drivers who were frequent speed violators were less favorable of ISA systems.

Although speeding is a frequent contributing factor in motor vehicle crashes, it is a relatively uncommon behavior among older adults. This is, perhaps, why so few studies of ISA systems have included older drivers. In general, ISA systems are not positively received by drivers and do not impact speeding behaviors unless they actively slow down vehicles that are speeding.

### Other systems

The final category of advanced in-vehicle technologies includes those that are not strictly collision avoidance or IVISs. Many of these systems can improve safety but may also function as a means to make driving easier or more comfortable. Here we review the following systems: adaptive cruise control; automatic crash notification; night vision enhancement; adaptive headlights; voice activated control; and drowsiness/fatigue warnings.

#### Adaptive cruise control

One of the earliest advanced in-vehicle technologies to be developed was adaptive cruise control (ACC) (de Winter et al. 2014). Regular cruise control requires the driver to brake if he or she gets too close to a forward vehicle. ACC systems, on the other hand, allow a driver to set a preferred headway, and a forward mounted sensor detects traffic in front of the vehicle, calculates the current headway, and interfaces with the throttle to change the vehicle’s speed to maintain a certain headway (Fancher et al. 1998; Hoedemaeker & Brookhuis 1998). ACC systems are designed primarily to make driving easier and as such are considered comfort and convenience technologies.

A study in England investigated behaviors and perceptions of 110 participants (age 18–73) using an ACC system in a simulator (Stanton & Young 2005). Participants drove simulated routes that varied in the levels of traffic with and without the ACC system activated. The study found that as compared to driving without the ACC system, use of the system decreased workload, stress, and situational awareness; reported frustration was higher for the ACC system in high levels of traffic; and there was no effect on locus of control or trust. The authors concluded that the ACC system served its purpose as a comfort and convenience technology, but cautioned that future designs should attempt to provide better situational awareness.

Some authors have argued that the ACC might have a negative impact on safety, primarily through a reduction in situational awareness, risk compensation, and a lack of understanding of the system’s functional limitations (de Winter et al. 2014; Hoedemaeker & Brookhuis 1998; Piccinini et al. 2014; Piccinini et al. 2015; Rajaonah et al. 2006; Seppelt & Lee 2007; Xiong et al. 2012). For example, a simulator study of 38 participants (age 25–60) who drove with and without ACC found that when driving with ACC, participants drove faster, with smaller minimum headways, and applied larger force to the brakes (Hoedemaeker & Brookhuis 1998). A study in Portugal compared drivers who were experienced users of ACC to people who were not (age 33–61) while driving in a simulator with and without ACC (Piccinini et al. 2015). During the driving trials, participants were presented with a vehicle that was stopped on the roadway in front of them requiring them to brake to avoid a collision (note that ACC is not designed to automatically stop the vehicle in this situation, as the systems are not forward collision warning systems). The study found that both experienced and inexperienced ACC users had increased risk of hitting the stopped vehicle as compared to not using ACC. Data revealed that many participants in both groups were not fully aware of how ACC would react to a stopped vehicle. This result is supported by studies that have asked users (mean age of 54–55) about limitations of their ACC system (Bato & Boyle 2011; Dickie & Boyle 2009).

A study of an ACC system installed in an instrumented vehicle used on a test-track reported similar concerns (Rudin-Brown & Parker 2004). This study investigated how ACC influenced driving among 18 drivers age 21-34. Participants drove an ACC-equipped vehicle behind a “surrogate” lead vehicle, while performing non-driving tasks. The study found that use of ACC reduced driver workload and increased response times in a hazard detection task. Drivers trusted the system even after a simulated failure, a condition in which trust should have been reduced. Drivers also had greater lane position deviation when using ACC. This latter result suggests that less attention was being paid to steering when using the system, but drivers were not crossing over lane boundaries. The authors suggested that use of ACC systems should be coupled with training on situational awareness and on how the systems operate.

The safety concerns of ACC have not been fully borne out in field tests, where people drive as they normally would in natural driving conditions. Fancher and colleagues (Fancher et al. 1998) furnished 108 participants (one-third age 60–70) with an ACC-equipped vehicle for their own use for up to 5 weeks. Collectively, these participants used ACC for more than 35,000 miles. Participants could turn the system on or off at any time, thereby self-selecting the traffic conditions under which the system was tested. Overall, participants used the ACC system in more than 50 % of their travel, primarily on limited access freeways. The older participants used the system the most frequently. The study found significantly higher deceleration rates while using ACC, suggesting greater intervention was required to avoid a collision. However, the authors noted that participants appeared to be using the system in a “supervisory” role, and waited to see if the system would resolve a forward conflict, with attention being drawn to the potential conflict by the deceleration from the ACC. If the system could not resolve the conflict quickly, then the driver applied the brake. The authors reported no crashes during the period of ACC use and, based on several analyses, concluded that ACC was safe. Participants overwhelmingly were comfortable with the system and found it highly attractive. Field tests of systems that coupled ACC and FCW technologies, most of which included older driver groups, have been conducted in both the US and Europe (Ervin et al. 2005; Kessler et al. 2012; Rakha et al. 2001) and have also found ACC to be safe and positively received.

A number of studies have investigated the use and perception of ACC systems among people (31–50 % of whom were older drivers, depending on the study) who have an ACC system in their personal vehicle (Strand et al. 2011; Eichelberger & McCartt 2014a; Cicchino & McCartt 2014; Eichelberger & McCartt 2014b; AAAFTS 2008). These studies have found that: ACC systems were used for a large majority of trips, most frequently on freeways, and older drivers used the system more than younger drivers; on ACC systems in which the headway can be a set by the user, older drivers tended to use the largest headway; one-third to one-half reported following vehicle less closely when using ACC; more than 90 % reported that they did not look away from the road more when using ACC; about 40 % reported that the ACC system made them a better driver; and about 40 % erroneously reported that the ACC system would help them avoid a collision with a stopped vehicle in the lane ahead.

A large literature exists for ACC systems and older drivers. This literature shows that older drivers value ACC systems for their comfort and convenience. Older drivers frequently used ACC systems and had lower levels of stress and workload while using the systems. On the negative side, however, ACC system use by older adults can result in reduced situational awareness, late braking for critical events, and overconfidence in the system. These negative effects are thought to arise from older drivers (and drivers of other ages) not fully understanding the situations under which ACC systems do and do not operate, such as automatically stopping the vehicle in the presence of a stopped lead vehicle. These negative effects are not generally seen in tests of ACC during natural driving, but the situations in which the ACC system would fail are rarely encountered in natural driving.

#### Automatic crash notification

Getting emergency services personnel to the exact scene of a crash quickly can raise the probability of saving lives and reduce the severity of injury outcomes. Automatic crash notification (ACN) systems (also called mayday systems) employ communication technology that can contact emergency medical services (EMS) personnel automatically within a few seconds after a crash (Hunt 2002; Williams 2002). The type of information transmitted to EMS varies depending on the system, but can include GPS location, vehicle information, and in some cases, data about the crash type and/or severity (Champion et al. 2003). ACN systems are usually triggered by an airbag deployment, but other crash sensors can be used (Walker et al. 2010). While not systems designed to impact driving or mobility, ACN systems can aid in saving lives by dispatching emergency assistance earlier than is normally possible.

Several studies have estimated the reduction in fatalities of ACN systems using actual crash data and assumptions about ACN system market penetration. An evaluation in Korea estimated that if all vehicles were equipped with ACN systems, 9–15 % of crash-fatalities could be prevented on Korean highways (Jeong et al. 2014). A study in Finland examined an ACN system and concluded that the system prevented 4–8 % of road fatalities (Sihvola et al. 2009). Another study estimated the effects of an ACN system employed across the US and reported that such a system could save 2–6 % of fatalities each year (Clark & Cushing 2002). Another US study using US data, reported that if an ACN system could notify EMS personnel within 1 min after a crash, the system could save up to 290 lives each year and reduce fatalities by 1.8 % within the first 6 h of a crash (Wu et al. 2013). An Australian study concluded that ACN in that country could prevent up to 10 % of passenger-vehicle fatalities each year (Lahausse et al. 2008). Other studies have also demonstrated the safety benefits and efficacy of ACN systems (Berryman 2004; Kanianthra et al. 2000). No research has directly considered the safety benefits of ACN systems for older drivers, but given the increased likelihood of frailty and fragility in older adulthood, it is logical to propose that ACN systems might have a greater safety benefit for older drivers and passengers.

Given the higher fatal crash rate and the increased likelihood of severe injury in older adulthood, any technology that can improve the chances of an older adult surviving a crash is recommended. ACN systems have been shown to reduce fatalities in crashes, although this has not been established among older adults. ACN systems operate automatically without any interaction from the driver.

#### Night vision enhancement

It is well-documented that one self-regulatory behavior engaged in frequently by older adults is avoiding driving at night due to difficulties with seeing at night (Baldock et al. 2006; Charlton et al. 2006; Molnar et al. 2013). When exposure is taken into account, there is some evidence that older drivers have higher nighttime crash rates than drivers in the middle-age group (see e.g., McGwin & Brown 1999; Massie et al. 1995; Stutts & Martell 1992). Technologies that help drivers see at night, called night vision enhancement (NVE) systems, have been proposed as a potential technology that can help older drivers while driving at night (Band & Perel 2007). NVE systems are designed to provide drivers with roadway information that is either difficult or impossible for the driver to obtain through direct vision, using infrared cameras to detect pedestrians, animals, signs, and other aspects of the roadway scene, intelligent image process, and video technology. This information is displayed on an in-vehicle video screen (Rumar 2002). Some systems also include a warning to alert drivers that an object, such as pedestrian, has been detected (Brown et al. 2010; Hankey et al. 2005).

Studies of NVE systems have utilized simulators, closed course test-tracks, and on-the-road studies, some of which included older adult groups of participants (e.g., Druid 2002; Gish et al. 2002; Gish et al. 1999; Ståhl et al. 1994; Sullivan et al. 2004; Raytheon Commercial Infrared and ElCAN-Teaxs Optical Technology 2000). Collectively, these studies have found that: drivers reported that they could intuitively interpret the displays; however, this ability seemed to be reduced when the display was not positioned above the steering wheel; NVE systems increased target detection distance by all drivers and the system only raised driver workload by a small amount; older drivers did not use the NVE system as frequently as younger drivers, possibly because of decreased divided attention capacity; the ability to detect pedestrians increased but only for younger drivers; generally system benefits were greater for younger drivers; older drivers reported being satisfied with NVE system; and older drivers did not think the system would result in a reduction of crashes. The actual safety benefits of NVE systems have not been established. Given that these study results suggest that NVE systems do provide some vision assistance with only small increases in workload, it is possible that they might improve safety for older drivers. More research is needed to establish the extent to which these systems do or do not improve safety for older drivers.

#### Adaptive headlights

As another way to improve vision while driving at night, systems have been developed to improve the effectiveness and functioning of the vehicle’s headlight system. These technologies, called adaptive headlight (AH) systems, involve a number of systems including ones that turn the highlights in the direction of a curve, automatically dim high-beam headlights in the presence of oncoming traffic, and control the direction and intensity of the headlight beam when opposite traffic is approaching (Band & Perel 2007). Collectively, AH systems are designed to improve nighttime driving visual capabilities for the driver of a vehicle and to reduce glare disability of drivers in other vehicles. The latter two systems are under development and little literature is available about the use or impacts of these systems.

A number of studies of AH systems that dynamically position the headlights in the direction of curves have been evaluated. Studies that have addressed the safety benefits of AH systems estimate that if fully-implemented in the US light-vehicle fleet, there would be an annual reduction in crashes of 2–5 % (142,000 crashes per year) and about 2700 pedestrian-related crashes per year could be prevented (Jermakian 2011; Mehler et al. 2014b; Sullivan & Flannagan 2007). Analysis of insurance data for vehicles equipped with AH systems, as compared to those without them, found a 5 to 10 % decrease in liability claims (Insurance Institute for Highway Safety 2012).

The impacts of AH systems on visibility of objects and pedestrians have been investigated on test-tracks and on the roadway. Sivak and colleagues (Sivak et al. 1994) compared an AH system to normal headlights (with 16 subjects, one-half of whom were age 62–72) on pedestrian detection, glare discomfort from the perspective of other drivers encountering the headlights, and thoughts about the systems after 30 kilometer trips in real traffic. The study found that: the AH system improved pedestrian visibility by 14 % for left-curves and 1 % for right-curves; discomfort glare was higher for the AH system on left-curves and lower on right-curves; participants thought the headlight movement was not smooth and took too long to return to straight ahead after curves; and there was no overall preference for either the AH or normal headlight systems. A European study included 22 participants (mean age 37) who drove several simulated trips through cities and rural areas over a 6-day period (Jenssen et al. 2007). All participants drove baseline trips with and without the AH system and then one-half used the AH system on the remaining trips while the other half used a standard headlight system. The study found no differences in driving patterns between groups or over time, with the exception of speed. All participants drove faster over time and participants using the AH system drove faster at night while in city traffic conditions.

Braitman and colleagues (Braitman et al. 2010) assessed the use of and opinions about AH systems among 290 owners of vehicles with such systems (20 % were age 61 or older). The study found that: 7 % of owners were not aware that the system was in their vehicle; 18 % thought the system improved visibility in general; 14 % thought the system was helpful for negotiating curves; 84 % reported that there was nothing they disliked about the system; 87 % preferred the AH system over standard headlights; and 77 % thought the system improved safety. When asked about changes in driving behaviors while using the system, 40 % reported that they were more willing to drive at night and 18 % reported that they were more willing to drive faster at night. No analyses by age group were presented.

Studies have estimated that AH systems can reduce nighttime crashes, particularly those involving a pedestrian. Although these studies do not provide estimates for the older driver population, to the extent that older people are driving at night, these benefits are likely to extend to this age group. Data with older drivers show that AH systems can improve the detection of objects and reduce the disability glare from oncoming traffic. There is also evidence that older adults may drive faster at night when using an AH system, which may or may not decrease safety. Of older drivers who have used the systems, about 1 in 5 report better nighttime visibility, a large majority prefer them to standard headlight systems, and most believe that they improve safety.

#### Voice activated control

Many advanced technologies, both in-vehicle and nomadic devices that link with in-vehicle technology, require input from the driver. Manual input can lead to various forms of distraction that could increase crash risk (Barón & Green 2006). Recently, technologies have been developed that utilize speech recognition algorithms that allow the driver to use voice commands to control various technologies such as adjusting the radio, processing email, phone dialing, and entering destinations into a navigation system. While not a self-contained technology, voice activated control (VAC) systems are designed to make interfacing with in-vehicle technologies easier and safer.

VAC systems have been compared to manual control systems in driving simulators on a variety of safety measures in several studies, none of which included older adults as participants (He et al. 2014; Itoh et al. 2004; Jenness et al. 2002; Lee et al. 2001; Maciej & Vollrath 2009; Strayer et al. 2013). These studies reported that: there was lower cognitive distraction for VAC systems than for manual control; both systems impaired driving performance significantly more when compared to driving without using either system; the manual system produced greater safety decrements in driving performance as compared to VAC systems; there was significant cognitive distraction when using VAC systems as compared to not using a VAC system; and reaction time was slower when using a VAC system. These studies suggest that VAC systems were safer than manual control but, as one set of researchers concluded: “Despite the appearance of safety…speech-based interface can distract drivers and undermine driving safety.” (Lee et al. 2001, pg. 639).

Test-track and on-road studies have also investigated safety and ease-of-use measures for VAC systems (Mehler et al. 2014b; Strayer et al. 2013; Chiang et al. 2005; Mehler et al. 2014a; Neurauter et al. 2012; Perez et al. 2011; Ranney et al. 2007; Reimer et al. 2013; Schreiner et al. 2004; Strayer et al. 2014). The results of the studies, some of which included older drivers as participants, found that: use of VAC systems in some instances produced significant levels of cognitive distraction; VAC systems had significant safety advantages over manual control systems on several measures; VAC systems were easier to use than manual entry; VAC systems as compared to manual entry systems were fast to use in some cases and slower in others; VAC systems improved driver performance when compared to manual systems; while using a VAC system, driving performance significantly declined while interacting with a range of in-vehicle technologies; and drivers liked VAC systems and would want them in their next vehicle. Results were generally the same among the older participants in these studies except: that older adults had greater difficulty using the VAC systems; and they experienced greater distraction and greater decrements in driving performance as compared to younger drivers.

As the number and complexity of advanced in-vehicle systems continue to grow, there will be a need to make interfacing with these systems as intuitive and simple as possible. VAC systems are a promising method for making interactions with in-vehicle and nomadic technologies easier and safer. One needs to keep in mind that simply engaging with several technologies while driving can increase workload whether it is through a manual or voice control system. The data show that VAC systems produce less cognitive distraction than manual controls, and that distraction is greater than interacting with passengers and engaging in many other non-driving activities.

## Discussion and conclusions

The intent of the paper was to synthesize the diverse literature on in-vehicle technologies and older drivers, knowing that there would be areas that were lacking sufficient research specifically on the age groups and/or technologies of interest. In the field of traffic safety, research designs are often different from the health research fields and one rarely finds randomized control trials. Therefore, we choose to not use meta-analytic (or other quantitative) techniques in this paper as these would not be appropriate for the preponderance of literature that exists in this area.

Research findings on 12 advanced in-vehicle technologies were reviewed with regard to how older drivers use and think about these technologies, and how the technologies can and do influence behaviors and safety outcomes. A summary of the findings and our conclusions of the potential overall value of each technology (judged as low, moderate, high, or too early to assess) for older drivers are presented in Table 2. Many of these technologies are available today in vehicles, while some of the technologies are under development and are expected to be available in the near future. We conclude that the following technologies could provide moderate to high benefits to older drivers: lane departure warning; forward collision warning/mitigation; blind spot warning; parking assist systems; navigation assistance; adaptive cruise control, if proper training is provided and it is paired with a FCW system; automatic crash notification; and adaptive headlights. For other technologies (intersection assistance; merging assistance; congestion warning; and drowsiness/fatigue waring), we conclude that they are too early in development to be able to assess the benefits for older drivers.

**Table 2:** Summary and conclusions for each technology

Technology	Use	Perceptions	Outcomes	Overall Value for Older Drivers
Lane Departure Warning/ Mitigation	• Frequent use • However, up to 22 % do not use system when available	• Considered helpful/useful, especially for long trips • Concerns about getting alerts soon enough • Small but non-trivial false alarm rates, usually in situations where lane markings poor/covered • Large percentage report wanting system in next vehicle	• Potential crash reduction of up to 30 % • Better lane keeping when distracted • Increased use of turn signals • Fewer lane excursions • Reduced stress	Moderate
Curve Speed Warning	• No information identified in literature	• Satisfaction rated as neutral • Some utility recognized	• No significant change in objective curve-taking behaviors • Some evidence of more appropriate speeds at night on closed course	Low
Forward Collision Warning/ Mitigation	• Nearly all drivers always keep the system on • Older drivers pick longer headways	• System rated positively • Some concerns about false alarms	• Faster reaction times to forward threats • Potential crash reduction of up to 20 % • Helps prevent crashes • Little negative behavior adaptation	High
Blind Spot Warning	• Frequent use • Use of system led to less frequent signal use	• Concerns about false alarms in bad weather • Some reported it to be distracting • Overconfidence in system	• Prevents crashes • Less frequent turning of head to check blind spot in 1/3 of participants • Increased situational awareness	Moderate (High when coupled with other collision warning systems)
Parking Assist: rearview display	• Most drivers always keep system on • 10–14 % of glances go to rearview display while backing • Warnings received at least once per week	• 95 % want system in next vehicle • 30 % report frequent unnecessary alerts when there is nothing behind vehicle	• Helps drivers notice obstacles behind them • Improves ability to fit squarely in parking spaces • 55 % reported system relieves stress • Combining backup video display with obstacle detection warnings enhances benefit	High
Parking Assist: cross traffic warning	• All drivers turn system on • All experience alerts	• Considered useful • Up to one-third report unnecessary alerts, mostly in bad weather or with stationary objects off to the side • Up to 15 % report failed alerts at least once, when another vehicle is approaching from behind very quickly • Reduces feelings of stress • Increases feelings of safety while backing up	• Helps prevent collisions when backing up • No changes in backing up behaviors	High
Parking Assist: semi-autonomous parking assistance	• No information identified in literature	• Positive ratings	• Reduced mental workload • Reduced stress • Improved parking behavior • Improved parking without the system	High
Navigation Assistance	• Frequent use • Take longer and have more difficulty than younger drivers learning to use system • Have more difficulty than younger drivers reading displays • More frequently use system with a “co-navigator” passenger	• Highly regarded	• Particularly helpful in wayfinding • More frequent travel during times and on roadways that would normally be avoided • Increased feelings of safety, confidence, attentiveness, and relaxation • Only minimal distraction reported	High
Intelligent Speed Adaptation	• Limited awareness of or experience with system	• Not positively received, especially for active systems	• No impact on speeding behaviors unless system actively slows down speeding vehicle	Low
Adaptive Cruise Control	• Frequent use • Full understanding lacking about situations under which system does and does not operate	• System valued for comfort and convenience • Overconfidence in system	• Lower levels of stress and workload • Reduced situational awareness • Late braking for critical events	Moderate (After proper training and/or if linked with FCW)
Automatic Crash Notification	• Does not require user input	• No information identified in literature	• High potential for fatal crash reduction	High
Night Vision Enhancement	• Used less frequently than by younger drivers	• Satisfaction with system • System not considered to result in crash reduction	• Provides some vision assistance with only small increases in workload • Increased target detection distance • System benefits greater for younger drivers	Low
Adaptive Headlights	• 7 % of owners not aware of system • System does not require driver input	• System considered to improve safety • Large percentage prefer system to standard headlight systems • More willing to drive at night with system	• 5–10 % decrease in liability claims • Potential 2–5 % crash reduction • Potential reduction of 2700 pedestrian-related crashes per year • 18 % report better visibility	Moderate to high
Voice Activated Control	• More difficulty using system than younger drivers • Greater distraction and decrements in driving performance compared to younger drivers	• System considered favorably • Most want the system in next vehicle	• Produces less cognitive distraction than manual controls • Produces greater distraction than interacting with passengers and engaging in other non-driving activities	Moderate

We began this paper by posing the question: Can advanced in-vehicle technologies help extend the period over which an older adult can drive safely? We answer this question with an optimistic “yes.” Some of technologies reviewed here have been shown to help older drivers avoid crashes, improve the ease and comfort of driving, and travel to places and at times that they might normally avoid. Other technologies show promise for providing benefits to older drivers and the development of these technologies continues. Although this report generally reviewed the technologies in isolation, the reality is that these technologies are being designed to work together as integrated in-vehicle systems (see e.g., Sayer et al. 2010). Designers of integrated systems strive to not only make using all of the technologies easier, but they also attempt to overcome the weaknesses of one system with the strengths of another.
